# Immunological pathogenesis of inflammatory bowel disease: focus on tissue resident memory T cells

**DOI:** 10.3389/fimmu.2025.1591584

**Published:** 2025-06-03

**Authors:** Jiayan Hu, Wenting Wang, Muyuan Wang, Chunye Wu, Yao Jiao, Yitong Li, Wenji Zhang, Chengtao Liang, Zhengdao Lin, Yitong Yu, Junxiang Li, Tangyou Mao

**Affiliations:** ^1^ Dongfang Hospital, Beijing University of Chinese Medicine, Beijing, China; ^2^ Beijing University of Chinese Medicine, Beijing, China; ^3^ Beitaipingzhuang Community Health Service Center, Beijing, China

**Keywords:** inflammatory bowel disease, tissue-resident memory T cells, immune balance, mechanisms, strategies

## Abstract

Tissue-resident memory T (T_RM_) cells are a type of tissue-restricted memory T cells with terminal differentiation and a memory function. They exist in mucosal tissues for a long period. In the absence of disease, T_RM_ cells promote essential inflammation, which reinforces the intestinal barrier and prevents bacterial translocation. However, in inflammatory or autoimmune environments, T_RM_ cells are hyperactivated. This heightened activity causes the host to release excessive pro-inflammatory cytokines, resulting in local immune imbalances and damage to the barrier, ultimately leading to tissue lesions. Numbers of studies have shown that T_RM_ cells play a crucial role in the development and progression of inflammatory bowel disease (IBD), suggesting that targeted regulation of T_RM_ cells homeostasis may be an important strategy for treating IBD. Here, we compiled the existing understanding of the role of T_RM_ cells in IBD, with particular emphasis on the associated mechanisms and approaches for targeting T_RM_ cells in IBD treatment. This review will serve as a foundation for a better understanding of IBD development and enhancing the effectiveness of clinical treatments for IBD.

## 1 Introduction

Inflammatory bowel disease (IBD), which includes Crohn’s disease (CD) and ulcerative colitis (UC), is a persistent inflammatory condition affecting the gastrointestinal tract, which is characterized by recurrent inflammation in local areas of the intestinal tract (1). In view of the chronic, early-onset and relatively low mortality of IBD, the prevalence of IBD has increased significantly over time with the increase of population aging. Studies utilizing the global burden of disease database indicate that the total number of cases is expected to rise from 298,412 in 1990-1994 to 490,887 by 2035-2039, with incidence rates consistently higher in men compared to women (2). The primary symptoms of IBD include abdominal pain, diarrhea, and hematochezia. While conventional treatment generally manages these symptoms, a complete cure remains elusive. A considerable number of patients may eventually necessitate surgical procedures and could encounter various complications, including extraintestinal manifestations, as well as disease-specific issues such as stenosis, fistulas, and abscesses (3). This has brought great pain to patients and affected their overall quality of life. Hence, it is crucial to investigate the underlying causes of IBD and create innovative and secure approaches for treatment.

The development of IBD is intricate and involves multiple factors, including genetic susceptibility, environmental factors, interactions between the microbiota and host immune system, and disrupted mucosal immunity (4). The intestinal immune system is a key factor that induces and maintains diseases (5–7). Despite genetic and environmental variations among patient populations, a dysfunctional immune response may ultimately lead to a common T cell-mediated inflammatory cascade that directly drives the development of IBD (8, 9). However, the mechanisms underlying abnormal intestinal immunity have not yet been fully elucidated. Various mucosal immune cells, including T helper cells (10), regulatory T cells (Treg) (11), innate lymphocytes (12–14), and macrophages (15), play a role in IBD development. However, the lifespan of most immune cells is relatively short, making it challenging to account for persistent immune abnormalities observed in IBD. Tissue-resident memory T (T_RM_) cells are generated from effector T cells and possess several key characteristics, including the abilities to continuously colonize tissues, secrete inflammatory cytokines, and self-proliferate, and a long lifespan. This aligns with the pathological features commonly observed in IBD (16). Recently, the important role of T_RM_ cells in IBD has been consistently validated (17). T_RM_ cells are terminally differentiated tissue-restricted memory T cells have a memory function and exist in mucosal tissues for a long period (18–20). T_RM_ cells are distinguished by their surface markers, including CD103, CD69, and CD49a, and absence of L-selectin (CD62L) and chemokine receptor 7 (CCR7). These features ensure the stable and long-term presence of T_RM_ cells within tissues. Their ability to contain and swiftly eliminate invading pathogens at the entry site is advantageous for the host, helping prevent tissue damage and systemic spread (21). Based on their phenotype and function, T_RM_ are mainly divided into CD8^+^ and CD4^+^T_RM_ cells. CD8^+^T_RM_ cells are usually located in the epithelial layer of the barrier tissue. Functioning as sentinels, they initiate antigen-specific immune responses upon reinfection. CD4^+^T_RM_ cells are typically situated beneath the epithelial layer, including within the basement membrane, and aggregate in lymphatic structures to enhance their interaction with antigen-presenting cells during reinfection (22). T_RM_ cells mediate a protective response against microbial infection (23). Even in a quiescent state, T_RM_ cells are capable of swiftly generating inflammatory mediators and stimulated surrounding tissues to upregulate defense mechanisms when stimulated (24, 25). Moreover, T_RM_ cells can directly eliminate infected cells by releasing cytotoxic molecules, such as perforin and granzyme (26). Some pathogen-specific CD4^+^and CD8^+^T_RM_ cells have powerful cytokine versatility, and produce high levels of interferon-γ (IFN-γ), interleukin-2 (IL-2), and tumor necrosis factor-α (TNF-α) (27, 28). T_RM_ cells are enriched on mucosal surfaces with a heavy pathogen burden, such as on the skin, lungs, and gastrointestinal tract (29, 30). Nonetheless, T_RM_ cells may undergo excessive activation in inflammatory or autoimmune contexts, leading to a ‘provocative state’ and resulting in pathogenic T_RM_ cells. The gut serves as a barrier organ with the greatest exposure to antigens, therefore, T_RM_ cells may play a vital role in facilitating localized immune responses specific to antigens in the gut (24), and may be involved in the development of IBD (31, 32). Furthermore, Zundler et al. (33) demonstrated that the presence of CD4^+^CD69^+^CD103^+^T_RM_ cells could predict disease onset and depletion of these T_RM_ cells limited colitis activity. While CD69 and CD103 are commonly used to identify T_RM_ cells, it is important to note that TRM populations are heterogeneous, and some subsets may not express these markers (34). However, in the context of IBD, most studies have focused on CD69^+^ and/or CD103^+^ T_RM_ subsets due to their prominence in mucosal tissues and established roles in immune surveillance and inflammation.

Here, we review the latest findings regarding the role of T_RM_ cells in the pathogenesis of IBD. We highlight that the imbalance between pro-inflammatory and regulatory T_RM_ cells, along with their intricate interactions within the immune network, constitutes a central pathological process. Therefore, we propose several therapeutic strategies aimed at targeting T_RM_ cells, including the regulation of their proliferation, activation, homing, and apoptosis, while simultaneously enhancing the function of Treg cells. Finally, we highlight the essential avenues for future research to further progress the field of IBD treatment.

## 2 Relationship between T_RM_ cells and IBD

### 2.1 Insights from clinical studies

#### 2.1.1 CD4^+^T_RM_ cells

Many clinical studies have confirmed that CD4^+^T_RM_ cells may be involved in the pathogenesis of IBD. Zundler et al. (33) observed an increase in the number of CD69^+^CD103^+^T_RM_ cells in the colons of patients with UC and CD. Importantly, they demonstrated that median flare-free survival in patients with high CD4^+^T_RM_ cell frequency was significantly shorter than in patients with low CD4^+^T_RM_ cell frequency (hazard ratio 3.39, 95% confidence interval 1.07-10.7). However, the exact mechanism still needs to be further elucidated. One possible mechanism could be related to the cytokine - mediated inflammatory cascade. CD4^+^CD69^+^T cells in the intestinal lamina propria of IBD patients produced significant amounts of pro-inflammatory cytokines, including IFN-γ, IL-13, IL-17A, and TNF-α. These cytokines play a role in tissue homeostasis and the innate immune response and have a profound impact on the intestinal microenvironment (35). For instance, IL-17A recruit neutrophils and promote the production of other pro-inflammatory mediators (36), which may disrupt the normal tissue homeostasis and contribute to the development of flares. Ohman L et al. (37) found that serum IL-17A levels of treatment-naive patients with UC reflect clinical disease severity at the onset of the disease and also predicted the disease course over the following 3 years. Consistent with this, Bishu et al. (32) analyzed the expression of related inflammatory factors in colon samples from patients with CD. They found that the levels of IFN-γ and IL-17A in CD4^+^T_RM_ were significantly higher in CD patients compared to the control group, and CD4^+^T_RM_ were identified as the primary subset of mucosal T cells producing TNF-α in these patients. High levels of CD103 in CD4^+^T cells correlate with increased production of pro-inflammatory cytokines and decreased expression of regulatory markers in individuals with UC (38). Yokoi et al. (39) found that CD103^+^CD4^+^T_RM_ exhibiting an inflammatory phenotype were significantly increased in the intestines of patients with CD, but not in those with UC. They identified distinct CD4^+^T_RM_ subsets with varying functions and transcriptional profiles in inflamed intestinal mucosa. These pathogenic CD4^+^T_RM_ subsets, characterized by their unique properties, are specifically enriched in CD and play a crucial role in coordinating the local inflammatory response.

#### 2.1.2 CD8^+^T_RM_ cells

Bottomis et al (31). revealed that CD103^+^CD8^+^T_RM_ cells in individuals with CD exhibited elevated expression of Th17-related genes, including IL-22 and IL-26, as well as genes encoding granzyme K, compared with the control group. The increased presence of Th17 T_RM_ cells throughout the small intestine in CD contributes to disease pathogenesis by inducing IFN-γ and subsequently promoting chemokine production in myeloid cells (40). Therefore, T_RM_ cells express more pro-inflammatory cytokines in patients with IBD, promoting the progress of IBD. In contrast, a recent study using single-cell RNA sequencing found that intestinal CD8^+^T_RM_ cells in UC undergo considerable transformation into an inflammatory state, which is partially linked to the elevated expression of the transcription factor Eomes. Eomes enhances the expression of various downstream target genes, including cytokines (IFN-γ), cytolytic effectors (Gzma) (41). This leads to increased secretion of pro-inflammatory cytokines, contributing to the exacerbation of local inflammation. Eomes is highly homologous members of the T-box family of transcription factors and are highly expressed by activated CD8^+^T cells (42). During murine skin T_RM_ cell differentiation following microbial infection, Eomes is initially upregulated; however, their expression decreases afterward to facilitate TGF-β signaling and support ongoing T_RM_ cell development (43). In summary, the available clinical evidence suggests an important association between T_RM_ cells and IBD pathogenesis, though their exact causal role requires further investigation. **Table 1** presents an overview of clinical research on T_RM_ cells and IBD.

**Table 1:** Overview of clinical studies of T_RM_ cells related to inflammatory bowel disease.

Object of study	Cohort description	Sample type	Marker analyzed	Outcome characteristics	Microbiota analysis approach	References
Adult	59 UC, 60 CD, and 1330 controls	Mucosal biopsy and peripheral blood samples	CD69^+^	Memory CD4^+^T cells are typically 2- to 4-fold more frequent in inflamed tissue from IBD patients compared with tissue from healthy controls.	Flow cytometry, Quantitative PCR	Hegazy AN et al. (2017) (17)
Adult	27 UC and 35 controls	Mucosal biopsy	αEβ7^+^	αEβ7 integrin expression on human colonic CD4^+^ T cells was associated with increased production of pro-inflammatory Th1, Th17 and Th17/Th1 cytokines, with reduced expression of regulatory T cell-associated markers.	Immunohistochemistry, flow cytometry and real-time PCR	Lamb CA et al. (2017) (38)
Adult	23 CD and 15 controls	Mucosal biopsy	CD69^+^	CD4^+^ T_RM_ cells are expanded in CD and more avidly produce IL-17A and TNFα relative to control cells.	Flow cytometry, Quantitative PCR	Bishu S et al. (2019) (32)
Adult	45 UC, 51 CD, and 14 controls	Mucosal biopsy	CD69^+^CD103^+^ CD69^+^CD103^-^	T_RM_ cells are increased in the gut of IBD patients and have a pro-inflammatory phenotype. CD4^+^CD69^+^CD103^+^ T_RM_ cells are driving disease flares in IBD.	Flow cytometry, Quantitative PCR	Zundler et al. (2019) (33)
Adult	4 CD and 3 controls	Mucosal biopsy and peripheral blood samples	CD103^+^, Klrg1^+^	CD103^+^CD8^+^T_RM_ cells from CD patients express Th17 related genes including CCL20, IL22, and IL26, which could contribute to the pathogenesis of CD	Cells Sorting and Flow Cytometry	Bottois H et al. (2020) (31)
Adult	7 UC and 9 controls	Mucosal biopsy and peripheral blood samples	not mentioned	In the setting of UC, there was a marked shift of clonally related CD8^+^T_RM_ cells towards an inflammatory state	scRNA-seq, scTCR-seq and scBCR-seq	Boland BS et al. (2020) (41)
Adult	27 CD and 10 controls	Mucosal biopsy	CD69^+^CD103^–^, CD69^+^CD103^+^, CD103 ^+^	During chronic inflammation, especially intraepithelial CD103^+^CD69^+^CD8^+^T cells displayed an innate proinflammatory profile with concurrent loss of homeostatic functions	Flow cytometry, RNA-seq	Lutter L et al. (2021) (151)
Adult	26 UC, 25 CD, and 26 controls	Mucosal biopsy and peripheral blood samples	CD103^+^	A subset of CD103^+^CD4^+^T_RM_, expressing CD161 and CCR5, were specific to CD patients and that this subset exerted cytotoxic activity.	Mass cytometry, scRNA-seq, fluorescence-activated cell sorting, TCR-seq, immunohistochemistry	Yokoi T et al. (2022) (39)
Adult	7 CD and4 UC	Non-inflamed small intestine tissue specimens	not mentioned	The elevated Th17 T_RM_ cells throughout the small bowel in CD, contributing to disease pathogenesis through IFN-γ induction and subsequent chemokine production in myeloid cells	scRNA-seq and paired scTCR-seq	Lee Y et al. (2024) (40)

### 2.2 Insights from animal studies

#### 2.2.1 CD4^+^T_RM_ cells

Consistent with the findings of human clinical studies, preclinical studies have demonstrated a correlation between the presence of T_RM_ cells and disease severity in animals with experimental colitis. DSS-induced colitis mice exhibited a notably increased number of CD4^+^ T_RM_ cells *in vivo* compared with that in control mice, and these levels markedly decreased after treatment (44). Zundler et al. (33) found that the knockout of tissue-specific transcription factors like Hobit and Blimp-1, two essential transcription factors for T_RM_ cells, inhibited the onset of colitis in several experimental mouse models (T cell transfer colitis, DSS-induced colitis, TNBS-induced colitis). Hobit (gene Zfp683), a homolog of Blimp1, suppresses genes involved in tissue egress (e.g., S1PR1, Tcf7, CCR7), thereby promoting retention in the small intestinal epithelium (45). Blimp1 itself downregulates Krüppel-like factor 2 (KLF2) and S1PR1 (46), which is critical for T_RM_ cell retention. Hobit and Blimp-1 are pivotal not only in regulating tissue retention, but also in controlling the cytotoxic functions of T_RM_ cells. In the absence of Blimp-1, the formation of T_RM_ cells in gut shows defects in the production of granzyme B (47).Mice with a dual genetic deletion of Hobit and Blimp-1 in CD4^+^ T cells showed a decrease in the production of pro-inflammatory cytokines, including IFN-γ, IL-13, and IL-17A, and displayed a reduced influx of granulocytes and macrophages. Furthermore, depletion of T_RM_ cells effectively prevented the onset of experimental colitis. These findings confirm that T_RM_ cells play a crucial role in driving intestinal inflammation (31). Another study found that the number of CD4^+^T_RM_ cells in DSS-induced colitis mice was significantly higher than that in normal mice, and the number of CD4^+^CD69^+^CD103^-^T_RM_ cells was positively correlated with the disease activity index (48). Knockdown of TIGIT, a regulatory gene of CD4^+^T_RM_ cells, reduced the number of CD4^+^CD69^+^CD103^-^T_RM_ cells in the colon tissue and reduced the level of IL-17A. Furthermore, the transfer of intestinal CD4^+^T_RM_ cells into RAG2^−^/^−^ mice also induces experimental colitis (49). This suggests that CD4^+^T_RM_ in the gut disseminate and cause local inflammation in UC. CD4^+^T_RM_ cells were present in C. rodentium-infected mice, showing significant enrichment in the intestines and serving as the primary source of IL-17A (50). Paired immunoglobulin-like receptor B regulates the survival of CD4^+^IL-17A^+^T cells. Loss-of-function inhibits the differentiation and growth of CD4^+^IL-17A^+^T_RM_ cells, thereby preventing the development of CD4^+^T cell-dependent colitis in a mouse model of T cell metastasis (35). Another study conducted in mice demonstrated that the insulin receptor present on gut T cells contributes to the differentiation of T_RM_ cells, particularly CD4^+^T_RM_ cells, by affecting the action of the Enhancer of Zeste Homolog 2 (EZH2). This process ultimately worsens intestinal inflammation by increasing the production of cytokines, such as TNF-α and IL-17 (51). **Table 2** presents an overview of the animal studies related to T_RM_ cells and IBD.

**Table 2:** Overview of animal studies of T_RM_ cells related to inflammatory bowel disease.

Object of study	Cohort description	Sample type	Marker analyzed	Outcome characteristics	Microbiota analysis approach	References
Wild-type (WT) and OT-II mice	Colitis model induced by gavage with Citrobacter rodentium	Feces and colon tissues	CD69^+^	*Citrobacter rodentium* induces CD4^+^ T_RM_ Cells. CD4^+^T_RM_ cells are the major source of mucosal CD4+T-cell-derived IL-17A and IFN-γ late after C. rodentium infection	Flow cytometry, Quantitative PCR	Bishu S et al. (2019) (50)
Hobit-Blimp-1 double knockout (DKO), Hobit knockout (KO), Rag1^−^/^−^ mice and WT mice	The initial CD4^+^ T cells from spleen wild-type mice were transferred to RAG1^−/−^ mice to establish a T cell metastatic colitis model;Rectal application of TNBS for 2 days to establish acute TNBS colitis;Mice were given DSS in drinking water for 7 days to induce colitis.	Colon tissues	CD69^+^	Hobit–Blimp-1 double knockout protects from several independent experimental colitis models. Depletion of T_RM_ cells protects from experimental colitis.	Flow cytometry, Quantitative PCR, Immunohistochemistry, immunofluorescence	Zundler S et al. (2019) (33)
TIGIT^−^/^−^ mice of C57BL/6 background	Mice were given DSS (2% (w/v)) in drinking water for 7 days to induce colitis.	Spleen and colon tissues	CD69^+^ CD103^-^, CD69^+^ CD103^+^	TIGIT deficiency protected mice from induction of experimental colitis by reducing IL-17A producing CD69^+^CD103^-^CD4^+^ T_RM_ cells.	Flow cytometry, Immunohistochemistry, scRNA-Seq Analysis	Chen B et al. (2022) (48)
C57BL/6mice	Mice were given DSS (2.5% (w/v)) in drinking water for 7 days to induce colitis.	Colon tissues	CD69^+^	The content of CD4^+^T_RM_ cells in the colonic lamina propria of the model group mice was significantly higher than that of the blank group	Flow cytometry	Wang et al. (2023) (44)
C57BL/6mice, EZH2fl/fl (WT) and EZH2fl/ fl CD4cre(KO)mice	Mice were treated with 3 cycles of DSS. Each cycle consists of 7 days of drinking water containing 2% DSS and 2 weeks of standard drinking water followed.	Colon tissues	CXCR6^+,^ CD69^+^	The INSR of intestinal mucosal T-cells could promote intestinal T_RM_ differentiation via EZH2 and exacerbate chronic colitis. CD4^+^T_RM_ was significantly increased in chronic colitis.	Flow cytometry, qPCR, Cytometric Bead Array	Li T et al. (2024) (51)

### 2.3 Insights from single-cell and spatial transcriptomic

Recent advancements in single-cell RNA sequencing (scRNA-seq) and spatial transcriptomics have provided unprecedented insights into the heterogeneity and compartmentalization of T_RM_ cells in IBD. For instance, Boland et al. (41) identified CD8^+^ T_RM_ cells localized to epithelial niches in UC patients, where they exhibit clonal expansion and Eomes-driven proinflammatory signatures, directly contributing to epithelial damage via IFN-γ and granzyme release. These cells were found to reside within specific epithelial niches, suggesting that they may contribute to local barrier disruption and tissue remodeling, which are central elements of IBD pathology. Similarly, Lutter et al. (52) demonstrated that intestinal CD4 T cells, including T_RM_ cells, exhibit distinct transcriptional profiles based on their anatomical location (epithelium vs. lamina propria), with significant changes observed in the epithelium during inflammation. This compartmentalization underscores the importance of understanding how T_RM_ cells interact with their local microenvironment to drive disease progression. In another study, Lee et al. (40) characterized Th17 T_RM_ cells in the non-inflamed intestinal tissue of CD patients using scRNA-seq and paired T cell receptor sequencing. They found that Th17 T_RM_ cells in CD exhibited a heightened expression of tissue-residency markers (ITGAE, ITGA1, and CXCR6) along with elevated levels of IL-17A, IL-22, CCR6, and CCL20. These cells also showed increased IFN-γ-related signatures, potentially linked to STAT1 activation, which could induce chemokines in myeloid cells and contribute to local inflammation. This finding highlights the role of Th17 T_RM_ cells in maintaining chronic inflammation in CD and underscores the need for targeted therapeutic strategies. These studies collectively emphasize the importance of integrating single-cell and spatial transcriptomic data to elucidate the mechanisms by which T_RM_ cells contribute to IBD pathogenesis. Understanding the specific transcriptional profiles and spatial distribution of T_RM_ cells can provide critical insights into their role in local barrier disruption and tissue remodeling, potentially leading to the development of more effective targeted therapies.

There are extensive connections between T_RM_ cells and IBD. However, T_RM_ cells exhibit inconsistent behaviors in different locations of the intestine and under different conditions (active or remission phases of the disease). A comparative study of different anatomical locations of CD8^+^T_RM_ cells in gut by John T. Chang’s research team has more comprehensively revealed the phenotypic and functional heterogeneity of CD8 ^+^T_RM_ cells in four intestinal compartments (53). Quantitatively, the order of intestinal CD8^+^T_RM_ cell numbers was found to be intestinal epithelial cells(siIEL) > Lamina propria cells of small intestine(siLP) > colonic lamina propria (cLP) > colonic epithelial cells (cIEL) by both microscopic quantification and flow analysis. In terms of phenotypic heterogeneity, the majority of siIEL CD8^+^T_RM_ cells express both CD69 and CD103, CD69 CD103 subset account differently in siLP, cIEL and cLP CD8^+^T_RM_ cells. All in all, the heterogeneity of intestinal TRM cells still needs to be further elucidated.

## 3 The role of T_RM_ cells in IBD

**Figure 1:**
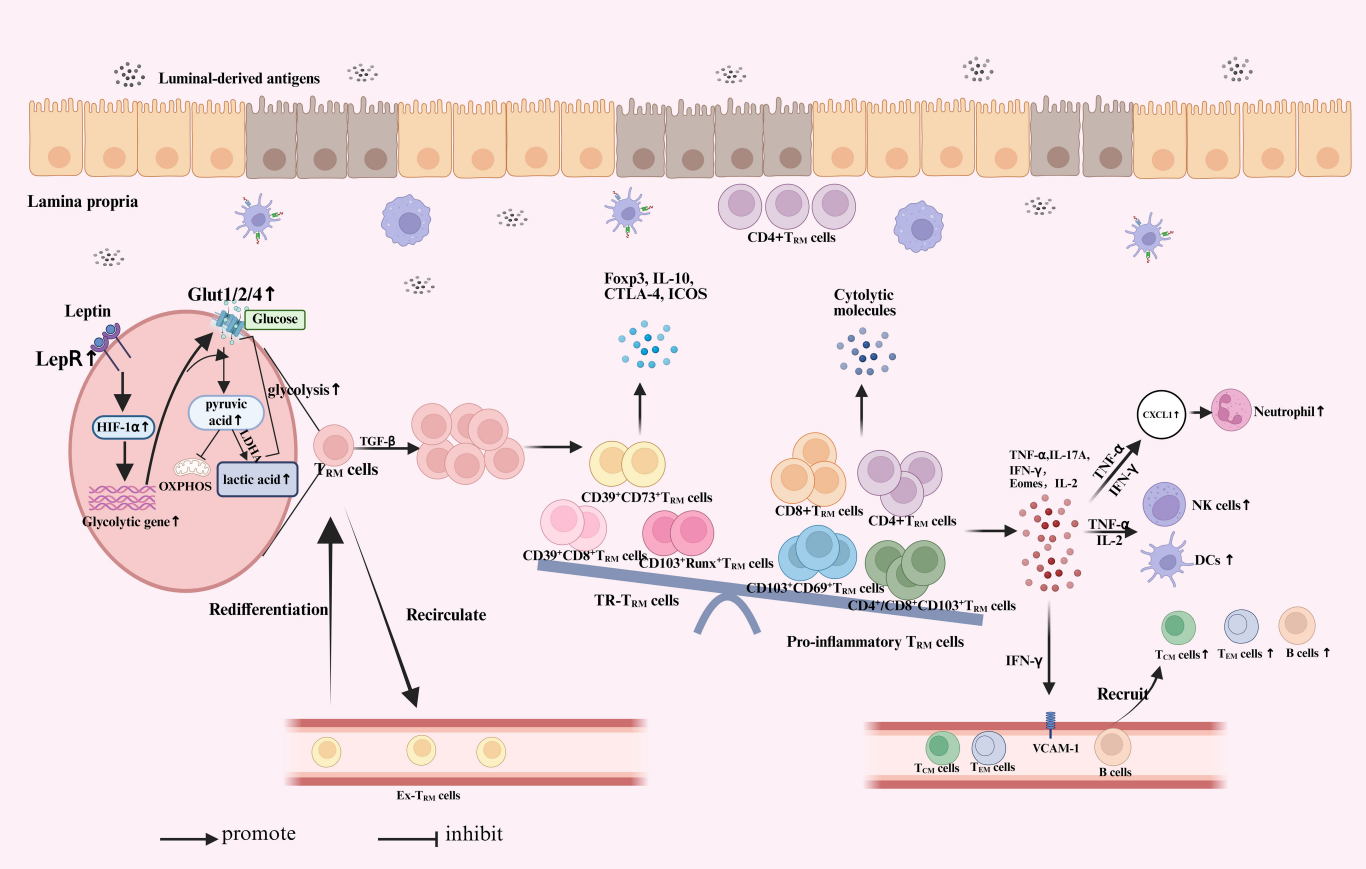
The role of T_RM_ cells in inflammatory bowel disease. When the intestinal epithelial layer is compromised, antigens from the intestinal lumen, including microbiota and pathogens, can penetrate the barrier and access the intestinal lamina propria. Under stress, T_RM_ cells upregulate LepR signaling and downstream HIF-1α expression to rapidly enhance energy production in response to antigen infection. This leads to a significant increase in Glut expression and glycolytic activity. With additional stimulation from TGF-β, pre-existing T_RM_ cells undergo local proliferation and play a key role in effective recall responses. Some of these T_RM_ cells may re-enter the circulation, showing a preference for migrating back and the potential to re-differentiate into T_RM_ cells. A fraction of T_RM_ cells undergo rapid proliferation and exhibit a greater propensity to differentiate into pro-inflammatory subsets, accompanied by the degradation of certain regulatory subsets. This change leads to an increased expression of inflammatory cytokines, chemokines, and cytotoxic granules. T_RM_ cells secrete proinflammatory cytokines, including IFN-γ, IL-2, and TNF-α, which activate natural killer (NK) cells and dendritic cells (DC). This process enhances neutrophil infiltration and recruits additional immune cells by increasing the expression of VCAM-1 on endothelial cells. Ultimately resulting in inflammatory infiltration and causing the development of IBD. At the same time, CD4^+^T_RM_ cells in the lamina propria are closely located to the intestinal epithelium, directly damage to intestinal epithelium. T_RM_, tissue-resident memory T cells; LepR, leptin receptor; HIF-1α, hypoxia-inducible factor 1 alpha; LDHA, lactate dehydrogenase; OXPHOS, oxidative phosphorylation; IFN, interferon; IL, Interleukin; DC, dendritic cells; TNF, tumor necrosis factor; TGF, transforming growth factor; NK, natural killer; VCAM, vascular cell adhesion molecule; ICOS, Inducible synergistic co-stimulation molecules; CTLA-4, Cytotoxic T-lymphocyte associated protein 4; Foxp3, Forkhead box protein P3.

While T_RM_ cells is primarily recognized for its protective role, pathogenic characteristics of T_RM_ cells have also been linked to a range of diseases, such as autoimmune conditions like vitiligo, psoriasis, and cutaneous lupus (54).The gut is continually exposed to external antigens, including microorganisms and dietary components. While the specific antigens associated with IBD remain largely unidentified, these antigens may trigger localized, recurrent inflammation (55). Therefore, it is reasonable to infer that the immunological recall function of T_RM_ cells, along with their ability to trigger local immune responses, contributes to the pathogenesis of IBD. Indeed, numerous studies have demonstrated that the generation and presence of T_RM_ cells contribute to the development of IBD. (**Figure 1**).

### 3.1 Imbalance between pro-inflammatory T_RM_ cells and regulatory T_RM_ cells

In IBD, a sustained inflammatory response is a key factor that contributes to disease progression. T_RM_ cells persist in the intestine for extended periods and are rapidly activated upon re-exposure to antigens, leading to a continued inflammatory response (21). This characteristic allows T_RM_ cells to play a vital role in the chronic inflammatory processes associated with IBD. In patients with IBD, there is a notable increase in the number of pro-inflammatory T_RM_ cells, coupled with a decrease in the number of regulatory subgroups. This shift may contribute to an immune imbalance in the intestine, exacerbating inflammation (56).

#### 3.1.1 CD4^+^T_RM_ cells

The pathological feature of IBD is excessive activation of innate immune cells in the intestine, leading to increased antigen presentation and secretion of inflammatory factors. T_RM_ cells further exacerbate this process by recognizing antigens and releasing cytokines (39). Multiple studies have confirmed that the number of CD4^+^ and CD8^+^T_RM_ cells in the intestines of patients with IBD significantly increases and produces large amounts of inflammatory factors, and the activation status of these cells is correlated with the severity of the disease (32, 33, 48). Moreover, CD4^+^T_RM_ cells in the lamina propria are located near the intestinal epithelium, and this spatial arrangement may further contribute to epithelial damage. In addition, T_RM_ cells may trigger or exacerbate inflammation by disrupting immune tolerance due to certain factors, such as genetics and the environment (57). Yokoi T et al. (39) has discovered that under pathological conditions, a specific group of CD103^+^CD4^+^T_RM_ cells, which express CD161 and chemokine receptor 5 (CCR5), are the primary source of pro-inflammatory cytokines in the lamina propria region of IBD. Expanded CD4^+^T_RM_ cells play an important role in the production of Th1 and Th17 cytokines by CD (39) and UC (38), highlighting the crucial role of this T_RM_ subset in the development of these conditions. In patients with IBD, microbiota-reactive CD4^+^T_RM_ cells also display a Th17-skewed phenotype, which may indicate the host’s protective response aimed at enhancing tissue integrity (17). In colon samples from patients with UC and CD, the number of CD103^+^CD69^+^cells increased compared to that of colon CD69^-^T cells, indicating increased mRNA expression of pro-inflammatory (IFN-γ, IL-17A, and TNF-a) (33). This suggests that the function of T_RM_ cells is closely aligned with that of Th1 and Th17 cells. Simultaneously, human CD4^+^T_RM_ cells were found to express specific molecules, including markers CCR8 and PPAR-γ, which are closely linked to pathogenic Th2 cells (58). Increased numbers of CD4^+^CD45RO^+^GPR15^+^ memory-type Th2 cells are found in the colon of patients with UC (59). On the other hand, in CD patients, CD4^+^T_RM_ cells exhibit unique effector properties distinct from conventional T cell subsets. These cells possess an innate-like activation mechanism that operates independently of requiring T-cell receptor (TCR) engagement, characterized by transcriptional enrichment in cytotoxic pathways (e.g., granzyme-perforin axis) and IL-12 responsiveness typically associated with NK cells. This distinctive molecular signature enables spontaneous release of cytolytic molecules (granzyme A) and inflammatory cytokines (IFN-γ), even in the absence of antigenic stimulation (39, 60).

#### 3.1.2 CD8^+^T_RM_ cells

In addition to CD4^+^T_RM_ cells, CD8^+^T_RM_ cells in patients with IBD also affect disease progression. Bottois et al. (31) found that CD103^+^CD8^+^T_RM_ cells in patients with CD express more Th17- (such as IL-22 and IL-26) and granzyme K-related genes than those in the control group. In UC, CD8^+^T_RM_ exhibit increased expression of IL-26 and granzymes, potentially contributing to disease pathogenesis (61). The subset of CD8^+^T_RM_ cells that express Eomes and are clonally expanded in UC are pro-inflammatory, displaying increased inflammatory traits (41).

#### 3.1.3 Tissue-resident Treg cells

T_RM_ cells possess regulatory functions, particularly in the human colon, where certain T_RM_ cells express markers characteristic of Tregs. Currently, the specific markers for Tissue-resident Treg cells (TR-Tregs), especially those in the intestine, remain unclear. It is generally believed that these cells express CD39, CD73, and Foxp3 under such characteristics (62). TR-Tregs are retained within non-lymphoid tissues and capable of producing cytokines, such as IL-10, which have the ability to quell pro-inflammatory responses from tissue-resident T cells and actively facilitate tissue repair (63). Burton OT et al. (64) found that tissue residency was generally short, on the order of ∼3 weeks, and extracted tissue Treg cells were tissue-agnostic on re-entry. And their results suggest that TR-Tregs operate under a different residency paradigm from T_RM_ cells, being characterized by slow percolation through multiple tissues in a pan-tissue adapted state. Intestinal GATA3^+^Helios^+^ and RORγt^+^ are two main subtypes of TR-Tregs (65). GATA3^+^Helios^+^ and RORγt^+^Tregs are mainly derived from thymus and play the key immunosuppressors during intestinal inflammation. Deletion of GATA3 in Tregs leads to the development of a spontaneous inflammatory disorder with impaired suppressive function, reduced Foxp3 expression, and the adoption of Th17 phenotypes (66). RORγt^+^ Tregs constitute the main colonic Tregs subset, promoted by the microbiota (64). RORγt enhances Foxp3 expression in colonic Tregs, partly by suppressing effector programs. When RORγt-deficient Tregs are transferred into Rag-/- mice with induced colitis, they are unable to provide protection against the disease because of their Th1-like effector characteristics and the resulting loss of suppressive function (67). Studies on IBD have revealed that CD4^+^αEβ7^+^ T cells from both healthy individuals and patients with UC exhibit decreased mRNA expression of regulatory T cell-related cytokines and surface molecules (such as Foxp3, IL-10, CTLA-4, and ICOS), when compared to CD4^+^αEβ7^-^ T lymphocytes (40). Analysis of the intestinal mucosa of patients with IBD showed a decrease in the CD8^+^T_RM_ expression of CD39 (62, 68). CD39 is known to degrade excess extracellular adenosine triphosphate (ATP) and adenosine diphosphate (ADP) into adenosine monophosphate, whereas intestinal extracellular ATP and ADP promote colitis (69). In addition, Treg function is mediated by CD39 (62) and a decrease in CD8^+^T_RM_ expression of CD39 may exacerbate colitis. A reduction in the number of CD39^+^CD8^+^T_RM_ cells has also been noted in children with active IBD (68). In mice with depleted circulating CD8^+^T cells, T_RM_ cells assume effector cell functions when re-challenged with a virus. These T_RM_ cells kill infected cells by releasing granzyme B and perforin, effectively controlling the pathogen independent of natural killer cells (70).

#### 3.1.4 Natural killer T cells

Natural killer T (NKT) cells have been shown to be tissue resident. NKT cells are a special subset of T cells that simultaneously express the TCR and surface markers of NK cells. They have been proven to be able to colonize barrier tissues such as the intestine and liver, and exhibit the typical residence characteristics of TRM cells (23). Resident NKT cells display a distinct pattern of integrins that is integral to their long-term tissue retention (71).Melsen JE et al. (72) found that lymphoid tissue-resident CD69^+^CD8^+^T_RM_ cells share a transcriptional and phenotypic profile with CD69^+^CXCR6^+^ lymphoid tissue (lt)NK cells by the use of published data. NKT cells exhibit both protective and harmful roles in inflammatory bowel disease (IBD). On one hand, when stimulated with α-galactosylceramide, NKT cells provide protective effects in theDSS-induced colitis model (73). Conversely, NKT cells can also contribute to detrimental inflammatory and immune responses in the gut, such as promoting oxazolone-induced colitis through the production of IL-13 (74).

Energy metabolism plays a crucial role in the immune function of T_RM_. Under aerobic conditions, normal cells generate energy through cytoplasmic glycolysis followed by mitochondrial oxidative phosphorylation. However, in hypoxic environments, such as during inflammation, cells shift their energy production to glycolysis, which does not require oxygen, to meet their energy demands (75). Activated effector T cells predominantly utilize glucose through the glucose transporter, Glut1. The expression of Glut1 is essential for glucose metabolism, proliferation, and production of inflammatory cytokines in effector T cells. Th1 and Th17 cells demand substantial amounts of glucose for aerobic glycolysis, which supports T-cell activity and generates the biosynthetic precursors necessary for cell growth and division. In contrast, Tregs do not depend on Glut1; they rely on oxidative metabolism for their energy needs and suppressive functions (76, 77). These findings indicate that different metabolic programs are necessary for the development of T cell subsets and these programs can be modulated *in vivo* to regulate the development of Tregs and pro-inflammatory T cells in inflammatory diseases. The leptin receptor (LepR) and hypoxia-inducible factor 1 alpha (HIF-1α) pathway play crucial roles in regulating glycolytic metabolism. HIF-1α activates the transcription of Glut1, Glut2, and Glut4, enabling the body to adapt to low-oxygen environments. Clinical studies have revealed abnormal upregulation of LepR and HIF-1α expression in patients with UC (78, 79). Wang et al. (42) found that the levels of LepR and HIF-1α genes in the colons of colitis mice were significantly elevated, along with abnormal overexpression of glucose transporters, such as Glut1. Additionally, the levels of key glycolytic enzymes, including hexokinase 1 and pyruvate kinase 2, were elevated compared with those in healthy mice. These findings suggested that the metabolic profile of colitis mice underwent reprogramming and shifted towards glycolysis. These results suggest that under hypoxic conditions, the glycolytic activity in the body is substantially increased. This increase may promote pro-inflammatory T_RM_ cell differentiation, while inhibiting the differentiation of TR-T_RM_ cells. This imbalance in the immune response may contribute to the development of intestinal inflammation.

In conclusion, the immune system is abnormally activated under the stimulation of various antigens, and the function of T_RM_ cells is transformed to Th1 and Th17 cells, resulting in an imbalance between pro-inflammatory T_RM_ and regulatory T_RM_. This will increase the expression of pro-inflammatory factors, such as TNF-α, IFN–γ, and IL-17, and reduce the expression of anti-inflammatory factors, such as IL-10, which will eventually induce local inflammatory response and cause or aggravate IBD.

### 3.2 Influence the behavior or function of other immune cells

T_RM_ cells influence local immunity and inflammatory responses by directly interacting with other immune cells, such as macrophages and dendritic cells (DCs), thereby promoting their activation and function and enhancing local immune responses (80). T_RM_ cells secrete pro-inflammatory cytokines, such as IFN-γ, IL-2, and TNF-α, which activate natural killer and DCs. They also promote the recruitment of additional immune cells by increasing the expression of vascular cell adhesion molecule-1 (VCAM-1) on endothelial cells, thereby boosting the local immune response (81). T_RM_ cells also recruit and activate other immune cells, such as neutrophils and macrophages, leading to local immune disorders and further aggravation of intestinal inflammation (53). Zheng et al. (82) show that CD4^+^T_RM_ cells enhance the expression of cytokines, IFN-γ and TNF-α, which may lead to increased chemokine CXCL1 expression. This process recruits neutrophils and contributes to the chronic recurrent inflammation observed in atopic dermatitis. Funch et al. (83) arrived at a similar conclusion, demonstrating that CD8^+^T_RM_ cells induce the production of CXCL1 and CXCL2 in the skin, leading to the recruitment of neutrophils and subsequently triggering allergic contact dermatitis. Additionally, Stelekati et al. (84) discovered that activated CD8^+^T cells enhance the expression of MHC-1 and the costimulatory molecule, 4-1BB, on mast cells in an *in vitro* co-culture environment. However, the mechanism underlying this effect, whether mediated by T cell-derived chemokines or direct cell-to-cell interactions, remains unclear and requires further investigation.

## 4 T_RM_ cell-based strategies in IBD treatment

Recently, T_RM_ cells have become crucial for the occurrence and progression of IBD, and a focal point of research. These unique immune cells play pivotal roles in the regulation of intestinal inflammation. They participate in the inflammatory response and affect the process and severity of the disease. With further research on the immune mechanisms underlying IBD, increasing evidence suggests that targeting T_RM_ cells may be a promising therapeutic strategy. Regulation of the activation and function of T_RM_ cells may help alleviate the symptoms and pathological changes associated with IBD. Currently, some drugs and treatment methods have shown efficacy in clinical trials, showing potential for IBD treatment (**Table 3**).

**Table 3:** Therapeutic targets against T_RM_ cells in IBD.

Strategy	Targeted pathways	Representative drugs	Mechanism	Adverse events	References
Regulating the Activation and Differentiation of T_RM_ cells	TNF-α	Infliximab	IgG1 anti-TNF antibodies	Allergic reaction, Cutaneous side effect, Neurological side effect, Dyspnoea, Arthralgia and/or myalgia,	Zelinkova Z (152)
		Adalimumab	IgG1 anti-TNF antibodies	Allergic reaction, Cutaneous side effect, Neurological side effect, Dyspnoea, Arthralgia and/or myalgia, Injection site reactions, Infectious side effect	Zelinkova Z (152)
		Golimumab	IgG1 anti-TNF antibodies	Gastrointestinal disorders, Infections and infestations,Respiratory, thoracic, and mediastinal disorders	Yu J (153)
Disrupt the migration and colonization of T_RM_ cells.	S1PR	Ozanimod	Suppress S1PR1 and decrease the migration of lymphocytes to tissues.	Hypertension, Upper respiratory tract infection, Gamma-glutamyltransferase increased, Anaemia, Back pain, Nasopharyngitis, Headache	Sandborn WJ (154)
		Etrasimod	Suppress S1PR1, decrease the migration of lymphocytes to tissues and decreases pro-inflammatory cytokines,	Infection, abnormal liver function, gastrointestinal reaction, allergy	Al-Shamma H (155)
	Integrin	Vedolizumab	blocking the binding of α4β7 to MADCAM1, thereby preventing T cells from homing to the gut	Nasopharyngitis, arthralgia, headache, nausea	Loftus EV Jr (101)
		Etrolizumab	affect both αEβ7 binding to E-cadherin and α4β7 binding to MADCAM1, thereby preventing T cells from homing to the gut	injection site erythema, arthralgia, headache	Sandborn WJ (102)
		PF-00547659	blocks α4β7 binding to the MAdCAM-1 ligand	Clostridium difficile infection, anal abscess and anal fistula	Vermeire S (109)
	TGF-β	The expression of TGF-β is significantly elevated in active IBD patients, particularly in the lamina propria.			Kanazawa S (118)
	CCR9	CCX282-B	inhibits CCR9- and CCL25-dependent chemotaxis	Abdominal pain,Diarrhoea,Nausea,Headache,Arthralgia,Pyrexia	Keshav S (119)
Inducing apoptosis of Pro-T_RM_ cells	PD-1	Excretory/Secretory Products From Trichinella spiralis Adult Worms Attenuated DSS-Induced Colitis in Mice by upregulating PD-1. PD-L1 blockade led to increased percentages of CD103 cells and greater per-cell CD103 expression levels in exhausted-like T_RM_ cells			Wang Z (121)
	IL-23	Ustekinumab	Targeting the p40 subunit of IL-12 and IL-23 to inhibit the activity of IL-23.	headache, nasopharyngitis, upper respiratory tract infection (URTI), nausea, abdominal pain, vomiting, arthralgia, pyrexia, and fatigue	Sandborn WJ (141)
Enhancement of the function of TR-Treg cells	Mtorc	Rapamycin	Inhibit the mTOR signaling pathway	skin eruptions, infection,gastrointestinal reaction	Mutalib M (156)

### 4.1 Effect of the activation and differentiation of T_RM_ cells

Cytokines, such as TGF-β, IL-6, and TNF-α, can promote T_RM_ cell differentiation (56). TNF signaling not only promotes the expression of acute-phase proteins, but also affects key cellular behaviors, including migration, proliferation, and cell death, in a manner that is highly dependent on the context. Elevated levels of TNF-α promote cell proliferation, differentiation, and the enhancement of adhesion molecules on endothelial cells, facilitating increased cell migration to the inflamed site (85). TNF-α inhibitors, such as infliximab, adalimumab, and golimumab are used for IBD treatment. They mainly trigger apoptosis of CD4^+^T cells *in vivo (* 86) by fostering and maintaining an anti-inflammatory IL-10^+^ phenotype, while also slowing down the activation, maturation, and proliferation of CD4^+^ T cells (87, 88). Atreya et al. (86) observed the occurrence of apoptosis in the lamina propria of six patients four weeks after initiating treatment with infliximab or adalimumab. By demonstrating a noteworthy surge in active caspase staining within the lamina propria, particularly in CD4^+^ T cells, they presented compelling evidence for the induction of T cell apoptosis by anti-TNF. In conclusion, TNF-α induces apoptosis in T cells, indicating its potential role in regulating the quantity and functional state of T_RM_ cells. This study offers a novel perspective on IBD treatment. However, a comprehensive assessment of the specific effects of TNF-α inhibitors when administered to humans, including their therapeutic efficacy and potential adverse reactions, requires further rigorous and systematic investigation for validation.

### 4.2 Interference of the migration and colonization of T_RM_ cells

To migrate to the intestine, primed T cells need to express specific migratory receptors. This involves the upregulation of CCR9, α4β7, CD103, and CD69, along with the downregulation of sphingosine-1-phosphate (S1P) receptor 1 (S1PR1) and CCR7 (89, 90).

#### 4.2.1 Upregulation of S1PR1 signaling

Sphingosine-1-phosphate (S1P) is a membrane-derived lysophosphatide signaling molecule that regulates the immune response. S1P1 is the most common S1P receptor because it is expressed in endothelial cells and lymphocytes (91, 92). CD69 is a reliable marker of tissue residency, and inhibits exits from lymphoid organs and peripheral tissues by counteracting S1PR1, which has traditionally been viewed as having its primary role in the establishment of T_RM_ cells (93). The expression of S1PR1 on T cells allows them to detect gradients of S1P concentration, facilitating the chemotactic migration of these cells and enabling the exit of T cells from lymphoid tissues (94). The CD69 surface marker is usually positive for intestinal T_RM_ cells, but its target, S1P1R, is weakly expressed (95). Therefore, S1P1 negatively regulates the development of T_RM_ cells. The interaction between S1P and S1P1 inhibits the outflow of lymphocytes from lymphoid organs by triggering the internalization and degradation of the S1P1 receptor to ensure that lymphocytes do not migrate to the tissues. This may impair the local production of intestinal T_RM_ cells (96). Drugs that directly act on the S1P1 pathway, such as ozanimod and etrasimod, are currently being studied for IBD treatement (97). Ozanimod has shown superior efficacy compared with that of the placebo in treating patients with moderate-to-severe active UC for both induction and maintenance therapy, and has received approval from the FDA and EMA for UC. Results from two separate phase 3 trials demonstrated that etosimod (2 mg daily) was effective and well tolerated as an induction and maintenance therapy in patients with moderate-to-severe active UC. In the ELEVATE UC 52 trial, the clinical remission rate at week 12 was 27.0% for the etrasimod group compared with 7.4% for the placebo group, and at week 52, the rates were 32.1% and 6.7%, respectively. Similarly, the ELEVATE UC 12 trial demonstrated that etrasimod treatment significantly outperformed the placebo in various measures, including endoscopic improvement, symptomatic remission, and mucosal healing (98).

#### 4.2.2 Targeted integrins

A research demonstrated that DCs from the Peyer Patches are especially effective in promoting gut-homing traits in T cells by increasing the expression of migratory receptors and integrin α4β7 (99). Additionally, the Listeria monocytogenes (Lm) infection model established by Sheridan et al. (20) confirmed that Lm infection and the migration of T cells to the intestinal lamina propria and epithelium were, at least partially, dependent on the expression of α4β7. CD103, predominantly expressed on intestinal CD8^+^T_RM_ cells (29), is a well-established marker for TRM cells, particularly those residing at epithelial surfaces. By interacting with E-cadherin, CD103 facilitates the establishment and retention of intestinal T_RM_ populations in both the small and large intestines (100). The interaction between integrins and their ligands governs the movement and retention of white blood cells in peripheral tissues, such as the intestine, and modulates the local inflammatory milieu. Therefore, integrin-targeting treatments, such as etrolizumab and vedolizumab, are emerging therapeutic targets for IBD (101, 102).

Etrolizumab selectively targets the β7 subunit of α4β7 and αEβ7 integrins. Sandborn et al. (102) completed a phase III clinical trial of etrolizumab for the treatment of patients with moderate-to-severe CD. They found that compared with the placebo, the proportion of patients with who achieved clinical remission and endoscopic improvement significantly increased in the etrolizumab-treated group during the maintenance period. This finding indicates that etrolizumab effectively treats UC, and its mechanism may involve regulating the inflammatory response and promoting mucosal healing (103). The mechanism of etrolizumab blocking αEβ7 and α4β7 integrin heterodimer in the treatment of IBD is explained by its impact on T_RM_ cells. *Post-hoc* analysis of phase II trials in UC suggests that patients with high CD103 expression are more likely to respond to etrolizumab (104). Furthermore, etrolizumab disrupts the retention of epithelial cells of αEβ7-dependent CD8^+^T cells in the intestine (105). Gonzalez-Vivo et al. (106) discovered that patients in clinical remission have a higher baseline concentration of CD8 α4β7^+^ memory T cells than those not in remission. Furthermore, they identified CD8 α4β7^+^ memory T cell subpopulations as early indicators of remission in response to vedolizumab treatment for UC. Fischer et al. (107) injected human T cells or PBMCs into the NSG (NOD.Cg-Prkdcscid Il2rgtm1Wjl/SzJ) mouse strain that lacks murine T cells, B cells and NK cells via the ileocolic artery. They demonstrated that vedolizumab selectively hindered the movement of Tregs from patients with UC in this model, without affecting the migration of effector T cells. And a meta-analysis shows that the overall safety of vedolizumab is comparable to that of infliximab (108).PF-547659 is a recombinant IgG-2 monoclonal antibody that targets MAdCAM-1 and inhibits the binding of α4β7 to its MAdCAM-1 ligand. Compared with the placebo group, the 12-week clinical remission rate of patients administered pf-547659 was significantly higher (109).

#### 4.2.3 Targeted TGF-β

In the small intestine of mice, TGF-β induces the upregulation of CD103 and downregulates KLF2 expression in CD8^+^ T cells, both of which facilitate the retention of T_RM_ cells in that region (110). TGF-β activates the integrin-linked kinase and protein kinase pathways, initiating inside-out signaling of integrins, which strengthens the interaction between CD103 and E-cadherin (111). In addition, TGF-β downregulates the expression of T-box transcription factors, Eomes and T-bet, with Eomes levels gradually decreasing during T_RM_ cell development, while low T-bet levels enhance the expression of the IL-15Rβ chain (CD122), which is essential for the survival and function of T_RM_ cells (112, 113). Wang L et al (114). found that TGF-β-dependent downregulation of T-bet expression is an early event in the differentiation of intestinal CD103^+^T_RM_ cells. Moreover, the deletion of T-bet can partially bypass the blockade of CD103^+^T_RM_ cells formation caused by the absence of TGF-β signaling, promoting the differentiation of CD103^+^T_RM_. However, it should be noted that these factors exhibit context- and dose-dependent regulators roles. Their functions may be changed depending on inflammatory conditions and cellular activation states. Taking CD8+ T cells as an example, after blocking the TGF-β signaling pathway, the expression of Eomes is significantly upregulated, while the expression of T-bet shows a downward trend, resulting in an increase in the Eomes/T-bet ratio. This is associated with the differentiation of memory T cells (115). In the TGF-β-dominated intestinal microenvironment, the coordinated suppression of these transcriptional regulators appears critical for stabilizing the tissue-resident program. Therefore, inhibiting the function of TGF-β is expected to reduce the intestinal residency of T_RM_ cells and affect their function. Elevated levels of TGF-β expression have been observed in patients with active IBD, particularly in lamina propria lymphocytes (116). It is worth noting that previous research by Monteleone G et al. (117) has shown that T cells from IBD patients have a higher Smad7 expression. Smad7 is an inhibitor of the TGF-β signaling pathway, which adds complexity to the role of TGF-β in IBD. Kanazawa et al. (118) observed an increase in the expression of TGF-β2 and TGF-β3 in the lamina propria lymphocytes of patients with UC and CD. However, to date, no TGF-β inhibitors have been reported for the treatment of IBD. Conversely, mongersen, which restores TGF-β signaling, has been reported to effectively relieve the clinical symptoms of CD. Hence, further research is required to reveal the intricate relationship between TGF-β and IBD, with the aim of enhancing comprehension of its function in the disease.

Certain medications can influence the homing of T_RM_ cells through other mechanisms. CCX282-B is a targeted orally-administered antagonist of the chemokine receptor, CCR9, which regulates the movement and activation of inflammatory cells within the intestine. Results from a randomized controlled trial revealed that a greater percentage of individuals achieved clinical remission (CDAI ≤ 150) in the CCX282-B group compared with that of the placebo group at the conclusion of the maintenance period (119).

### 4.3 Induced apoptosis of pro-T_RM_ cells

Notably, CD4^+^and CD8^+^T_RM_ cells also express a range of surface molecules associated with the suppression of T cell activity, such as PD-1 (21). This serves as a crucial marker of T cell depletion linked to chronic infection and functional hypo responsiveness in tumors (120). The presence of PD-1 may limit the ability of T_RM_ cells to contribute to the tissue pathology. Research on lung infections revealed that PD-1 activation constrains the inflammatory action of T_RM_ cells. However, blocking PD-1 could enhance the proliferation and restoration of T_RM_ cells (121). Wang et al. (122) also discovered that excretory or secretory products from Trichinella spiralis adult worms (AES) exhibit a common property of mitigating DSS-induced colitis and this beneficial effect seems to be closely associated with an increase in PD-1 expression. Conversely, PD-1 deficiency compromised the therapeutic potential of AES in ameliorating DSS-induced colitis, highlighting the crucial role of PD-1 in mediating this therapeutic outcome. Blocking PD-L1 results in a high proportion of CD103^+^ cells and elevated CD103 expression levels per cell in exhausted T_RM_ cells (121). The classic anti-PD-1 antibody, nivolumab, induces colitis, which is called immune checkpoint inhibitor-associated colitis (123). Reschke R et al (124). studied colon biopsies from patients with immune checkpoint inhibitor-associated colitis compared with those from healthy people using immunofluorescence, spatial transcriptomics, and RNA *in situ* hybridization. They found that immune checkpoint inhibitor-associated colitis are dominated by T_RM_ cells and Th1/Tc1 cytokines. Emerging evidence suggests that PD-1 may differentially regulate T_RM_ subsets in barrier versus tumor tissues. In the gut, PD-1 signaling likely restrains pro-inflammatory T_RM_ activation to maintain mucosal tolerance (122). Blocking PD-1 could disrupt this balance, releasing a large amount of pro-inflammatory cytokines, leading to colitis. Conversely, in tumors, PD-1 inhibition may preferentially enhance anti-tumor T_RM_ cytotoxicity (125). This tissue specificity and the bidirectional nature of T_RM_ functions may explain why PD-1 blockade enhances T_RM_-mediated tumor immunity while triggering colitis.

IL-23 plays a vital role in the activation of T_RM_ cells. Mechanistically, IL-23 signaling through its heterodimeric receptor (IL-23R/IL-12Rβ1) activates STAT3 phosphorylation (pSTAT3), which drive their commitment to a Th17-like pathogenic subset—a process critical for the production of IL-17A and IL-22 (126), which in turn enhance epithelial permeability and inflammatory infiltration in the gut (127). Whitley SK et al. found that Local IL-23 is required for proliferation and retention of skin-resident memory Th17 cells and Administration of anti-IL-23R antibody to mice resulted in loss of CD69^+^ CD103^+^ tissue resident memory Th17cells from skin (128).Yen et al. (129) presented data indicating that IL-23-induced activation of T_RM_ cells contributes to chronic intestinal inflammation. Specifically, CD4^+^ T_RM_ cells are the key targets of IL-23 in the progression of chronic intestinal inflammation. Their findings demonstrated that IL-23 enhances the production of IL-6 and IL-17 by memory-activated T cells, and inhibiting IL-6 and IL-17 can improve IBD. While IL-17 inhibition holds theoretical potential for IBD treatment, clinical trials targeting IL-17A (e.g. secukinumab, ixekizumab) have yielded mixed results. Secukinumab (anti-IL-17A) and ixekizumab (anti-IL-17A) demonstrated efficacy in psoriasis and ankylosing spondylitis, yet are ineffective in IBD and may even trigger IBD in patients (130, 131). A study based on literature and database analysis found that IL-17 inhibitor treatment is associated with exacerbation and new onset of IBD and colitis. Patients who receive IL-17 inhibitors (secukinumab, ixekizumab, brodalumab) may experience obvious abdominal pain, diarrhea, and bloody diarrhea (132). These results may be attributed to the pleiotropic effects of IL-17A and heterogeneity of Th17 cells (133). On the one hand, IL-17A induces inflammation but also promotes intestinal epithelial barrier function and repair (134), and participates in an autoregulatory loop to limit the pathogenicity of Th17 cells (135). The plasticity of Th17 cells, influenced by various factors including T cell-polarizing cytokines and the inflammatory tissue environment, is crucial in maintaining gut mucosal homeostasis. On the other hand, there are two different types of Th17 cells: pathogenic and homeostatic. Pathogenic Th17 cells drive tissue damage by secreting pro-inflammatory cytokines such as IL-17A, IL-17F, GM-CSF, and IFN-γ. In contrast, homeostatic Th17 cells secrete IL-10, IL-22, and antimicrobial peptides to maintain the integrity of the mucosal barrier, promote epithelial repair, and inhibit excessive inflammatory responses (136). For example, in patients with psoriasis, a significantly elevated level of IL-17A has been observed in the lesional skin area (137). In the skin of healthy individuals and the non-lesional skin area of psoriasis patients, a small number of homeostatic Th17 cells are present, and their IL-22 may be involved in normal epidermal renewal (138, 139). Therefore, IL-17A inhibitors may worsen IBD by indiscriminately blocking both pathogenic T_RM_-Th17 activity and protective IL-17A-mediated barrier repair. And the limited effectiveness of drugs targeting IL-17A may be linked to the presence of ex-Th17 cells that switch to producing IFN-γ (140). In contrast, drugs targeting the upstream factor IL-23 (such as ustekinumab) indirectly regulate the level of IL-17 by inhibiting the differentiation of Th17 while preserving its protective functions, thus demonstrating better efficacy and safety in the treatment of IBD. Medications that target IL-12/IL-23, which are currently approved or are undergoing clinical trials for treating IBD, consist of fully human monoclonal antibodies that specifically target the p40 subunit shared by IL-12 and IL-23. Ustekinumab and briakinumab are examples of these drugs. In particular, ustekinumab has been highlighted as an effective treatment option. Ustekinumab is well tolerated, and effective in inducing and sustaining remission in patients with moderate-to-severe IBD who respond positively to the initial treatment, with no significant adverse effects or events reported (141).

### 4.4 Enhancement of the function of regulatory T_RM_ cells

**Figure 2:**
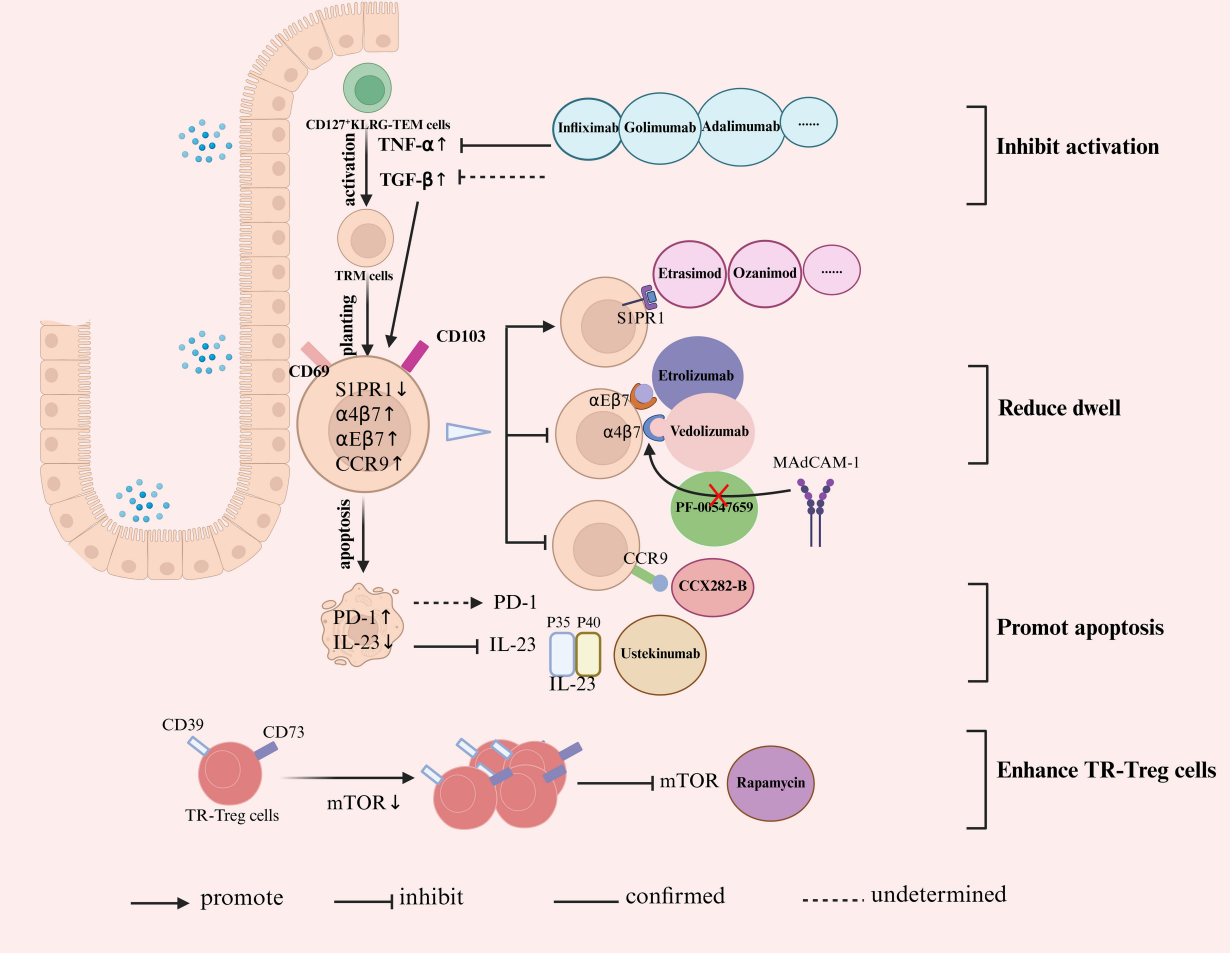
Targeted therapeutic strategies employing T_RM_ cells in the treatment of IBD. CD127^+^KLRG^-^T_EM_ cells can be activated into T_RM_ cells in reaction to TNF-α and TGF-β. The inhibition of TNF-α activity (using Infliximab, Golimumab, and Adalimumab) effectively suppresses the activation of T_RM_ cells. After activation, T_RM_ cells migrate from the circulation to the intestinal mucosa by upregulating the expression of αEβ7, α4β7, and CCR9 while downregulating S1PR1 expression. Therefore, downregulating the expression of CCR9 (CCX282-B), αEβ7 (Etrolizumab), and α4β7 (Vedolizumab), inhibiting the binding of MAdCAM-1 to α4β7 (PF-00547659), and upregulating S1PR1 expression (Etrasimod, Ozanimod) can reduce T_RM_ cell homing. The upregulation of PD-1 and the reduction of IL-23 (Ustekinumab) will promote the apoptosis of T_RM_ cells. Finally, Rapamycin boosts the differentiation, proliferation, and distribution of Treg cells by inhibiting mTOR.

The high number of Tregs present at the site of inflammation are still unable to protect against IBD. This indicates a potential compromise in Treg function in these patients (142). Conventional therapies for IBD exert beneficial effects on Tregs. Treatment of patients with UC using aminosalicylates or glucocorticoids elevate the frequency of CD4^+^CD45RO^+^CD25^+^T cells in the peripheral blood, a subpopulation that is abundant in Tregs (143). However, these methods potentially increase the risk of infection and lack specificity for exclusively targeting Tregs. Currently, therapies for IBD that target Tregs can be divided into two primary categories: cell-based treatments, which involve the transfer of *in vitro* expanded or stimulated Tregs to patients, and pharmacological approaches, which are designed to modify Tregs within the body (144). In a phase 1/2a clinical trial (145), ovalbumin-specific Tregs were effectively isolated from the peripheral blood mononuclear cells of patients with CD. These isolated cells underwent *in vitro* culture expansion before being intravenously reinfused into patients. Importantly, during the fifth and eighth weeks of the study, 40% of the participants showed a notable decrease of 100 points in their Crohn’s Disease Activity Index score. However, the transferred Tregs may fail to accurately migrate and localize within inflamed target tissues. An alternative strategy involves the use of drugs that modulate Tregs in the body. Janyst et al. (146) evaluated the effectiveness of different pharmacological agents believed to have immunomodulatory effects on Treg development. The researchers observed that rapamycin and prednisolone successfully increased the number of CD4^+^CD25^high^Foxp3^high^ cells and enhanced the expression of Foxp3 in Treg cells (recognized as CD4^+^CD25^high^Foxp3^high^ cells). Additionally, both medications extended the phenotypic stability of Tregs and stimulated the production of fully functional Tregs in functional assays. Rapamycin inhibits the glycolysis pathway and enhances the differentiation, proliferation, and distribution of Tregs, while inhibiting the formation of Th17 cells by targeting mTOR signaling. This process aids in reinstating the equilibrium between Th17 and Treg development, ultimately contributing to the treatment of IBD (147, 148). Rapamycin also increases the abundance of beneficial bacteria and decreases the abundance of harmful bacteria, extending experimental colitis (149). Goldberg R et al. (150) measured levels of the integrin α4β7 on Treg cells isolated from peripheral blood or lamina propria of patients with CD and healthy individuals (controls), and intervened with Treg cells *in vitro* using rapamycin. They found that incubation of patients’ ex vivo expanded Treg cells with rapamycin induced expression of α4β7, which might be developed for treatment of CD. Overall, the technology for utilizing Tregs to counteract T_RM_ cells in IBD treatment is still in its early stages. To develop novel therapies that rely on Tregs, researchers must first delineate the characteristics of Tregs throughout disease progression and reveal the mechanisms that hinder their suppressive function in IBD. These insights will facilitate the development of therapeutic strategies aimed at increasing the quantity, efficacy, and stability of Tregs. **Figure 2** illustrates a targeted therapeutic strategy using T_RM_ cells for IBD treatment.

## 5 Conclusions and perspectives

In conclusion, the intricate relationship between T_RM_ cells and IBD has been progressively revealed, highlighting the potential of T_RM_ cells as biomarkers and therapeutic targets. The chronic inflammatory characteristics of IBD are significantly influenced by the activation and function of T_RM_ cells within the intestinal mucosa. Although T_RM_ cells play a vital role in protecting the immune system from pathogens, dysregulation of these cells can result in excessive inflammation and tissue damage in IBD. The heterogeneity of T_RM_ cells and their plasticity in response to the intestinal microenvironment underscore the complexity of their role in IBD pathogenesis.

Despite these promising insights, several limitations and challenges remain. Firstly, the functions and mechanisms of action of T_RM_ remain unclear. The strategies for targeting T_RM_ cells to treat IBD discussed in the text remain merely theoretical possibilities. There is still a lack of direct evidence to prove their effectiveness, and the underlying mechanisms also await further clarification. Secondly, the heterogeneity of T_RM_ across patients and within the same patient at different disease stages complicates the development of targeted therapies. Targeting T_RM_ cells may disrupt the immune system balance. Finally, over-inhibition of T_RM_ cell activity may increase the risk of infections or adverse reactions beyond the gut. Future studies should elucidate the development, function, and regulatory mechanisms of T_RM_ cells to establish a robust theoretical foundation for targeted therapies. Additionally, understanding the role of T_RM_ cells in IBD will enhance our understanding of disease pathogenesis. This investigation may involve the identification of specific markers, transcription factors, and signaling pathways associated with T_RM_ cells, as well as their contribution to intestinal inflammation. The apparent contradictions between studies may reflect methodological differences in T_RM_ identification rather than true biological discrepancies. Studies examining different marker combinations (e.g., CD69 vs CD103) likely capture distinct T_RM_ subsets with divergent functions. Standardized multi-parameter phenotyping will be essential for future research.
